# Dental Materia Medica and Therapeutics

**Published:** 1877-03

**Authors:** James Stocken


### DENTAL MATERIA MEDICA AND THERAPEUTICS.

BY JAMES STOCKEN, D. D. S., R. C. S.

ASSISTANT DENTAL SURGEON TO THE NATIONAL DENTAL HOSPITAL, LONDON.

*Prepared by desire of the Medical Committee of the National Dental Hospital.*

By materia medica we mean the consideration of those substances employed in the treatment of diseases.

Therapeutics, the use and effect of those substances or medical material in disease.

All medical materials act either through the agency of the mind or directly on the body, and may be designated, mental and coporeal remedies;

1. Mental Remedies.—*a.* External. *b.* Internal.
   External.—Smell—taste—hearing—vision—touch.
   Internal.—Feeling—intellect—reasoning.
2. Physical Remedies (imponderable).
   Light (vital stimulus).
   Heat (vital stimulus).
   Cold (depressant and sedative, diminishing vital action).
   Electricity (stimulant to all senses).
   Magnetism.
3. Hygienic Remedies.—Food—climate—exercise,
4. Mechanical and Surgical Remedies.
5. Pharmacological Remedies.

Pharmacology or materia medica may be sub-divided into three departments:

1. Pharmacognosy treats of the origin, properties, varieties, quality and purity of *unprepared* medicines or simples.

2. Pharmacy treats of the collection, preparation, preservation and dispensing of medicines.
3. Pharmacodynamics and Therapeutics treat of the effects, uses and administration of medicines in the cure of diseases.

General Pharmacology may be thus viewed:

1. Modes of ascertaining effects of medicines.
2. Active forces of medicines.
3. Changes which medicines undergo in the organism.
4. Physiological effects of medicines.
5. Therapeutical effects of medicine.
6. Parts to which medicines are applied.
7. Classification of medicines.

Modes of ascertaining effects of medicines:

1. The sensible qualities of medicines.
2. The natural-historical properties.
3. The chemical properties.
4. The dynamical properties.

Active forces of medicines:

1. Physical.
2. Chemical.
3. Dynamical.

Changes which medicines undergo in the organism. These changes are either physical, chemical, or both.

Physiological effects of medicines may be physico-vital, chemico-vital, or purely vital.

The circumstances modifying the effects of medicines, may either relate to the condition of the medicine or the organism.

As regards the medicine:

1. State of aggregation.
2. Chemical combination.
3. Pharmaceutical mixture.

Relating to organism.—Age—sex—mode of life—occupation—habit—diseased condition of the body—climate—mind—race—temperament—idiosyncrasy—tissue or organ.

*Therapeutical effects of medicines.*—The effects produced on disease by the influence of medicines are denominated herapeutical, and are in their action direct or indirect.

There are three methods of employing medicine against disease, denominated:

1. Antipathic—consists in employing medicines which produce effects of an opposite nature to the symptoms of the disease.
2. Homœopathic—employing medicines capable of producing effects similar to the one to be removed.
3. Allopathic or hetropathic—employing medicines which give rise to phenomena altogether different or foreign (neither similar nor exactly opposite) to those of the disease.—*Review of Dental Surgery.*

